# Supplementary material for: Effect of the COVID-19 Pandemic on Suicide Mortality in Brazil: An Interrupted Time Series Analysis
Source: Int J Environ Res Public Health. 2025 Jan 21;22(2):138. doi: 10.3390/ijerph22020138 (PMC11855535; doi:10.3390/ijerph22020138)
Supplement: Supplementary file 1 [file ijerph-22-00138-s001.zip › Table S1.pdf]

Table S1. Effect of the Covid-19 pandemic on suicide mortality rates per 100,000 men, estimated through interrupted time series analysis and by comparing the periods January 2017 to February 2020 with March 2020 to December 2023, Brazil,2024.

Variables	Behavior after the intervention	Initial exploratory model without serial autocorrelation terms			Final model with n serial autocorrelation terms		
		Coefficient	Standard Error	Durbin Watson-test[c]	Coefficient	Standard Error	AR-terms[d]
Locality	Brazil[a]						
	Level change	-0.033	0.023	0.0205	-0.033	0.003	p=1
	Trend change	0.004	0.000		0.004	0.000	
	North[b]						
	Level change	-0.116	0.054	0.9285	DNA[e]	DNA[e]	DNA[e]
	Trend change	0.007	0.001		DNA[e]	DNA[e]	DNA[e]
	Northeast[b]						
	Level change	0.007	0.037	0.0039	0.0100	0.0070	p=1
	Trend change	0.004	0.001		0.0040	0.0000	
	Southeasta						
	Level change	-0.028	0.032	0.006	-0.029	0.007	p=1
	Trend change	0.004	0.001		0.004	0.0000	
	South[b]						
	Level change	-0.046	0.033	0.0496	-0.046	0.003	p=3
	Trend change	0.004	0.001		0.004	0.0000	
	Midwesta						
	Level change	-0.061	0.045	0.1112	DNA[e]	DNA[e]	DNA[e]
	Trend change	0.006	0.0010		DNA[e]	DNA[e]	DNA[e]
Age groups (years)	10 to 14 years[b]						
	Level change	0.133	0.178	0.191	DNA[e]	DNA[e]	DNA[e]
	Trend change	-0.001	0.004		DNA[e]	DNA[e]	DNA[e]
	15 to 19 years[a]						
	Level change	-0.022	0.069	0.001	-0.003	0.023	p=2
	Trend change	0.003	0.001		0.003	0.000	
	20 to 39 years[b]						
	Level change	-0.110	0.030	0.001	-0.113	0.010	p=2
	Trend change	0.007	0.001		0.007	0.000	
	40 to 59 years[a]						
	Level change	0.004	0.028	0.176	DNA[e]	DNA[e]	DNA[e]
	Trend change	0.003	0.001		DNA[e]	DNA[e]	DNA[e]
	60 or more years[a]						
	Level change	0.068	0.039	0.579	0.065	0.005	p=2
	Trend change	0.000	0.001		0.000	0.000	
Methods	Firearm[a]						
	Level change	0.112	0.053	0.904	DNA[e]	DNA[e]	DNA[e]
	Trend change	0.004	0.001		DNA[e]	DNA[e]	DNA[e]
	Self-poisoning[a]						
	Level change	-0.282	0.349	0.571	DNA[e]	DNA[e]	DNA[e]
	Trend change	0.005	0.007		DNA[e]	DNA[e]	DNA[e]
	Hanging, strangulation and suffocation[a]						
	Level change	-0.020	0.024	0.010	-0.019	0.004	p=2
	Trend change	0.005	0.000		0.005	0.000	

**Race/skin color**	White[a]						
	Level change	-0.074	0.029	0.074	DNA[e]	DNA[e]	DNA[e]
	Trend change	0.004	0.001		DNA[e]	DNA[e]	DNA[e]
	Black[a]						
	Level change	0.011	0.028	0.012	0.015	0.005	p=3
	Trend change	0.005	0.001		0.005	0.000	

[a]Interrupted time series in which the quasi-Poisson model with seasonality showed a better fit to the data compared to the model without seasonality; [b]Interrupted time series in which the quasi-Poisson model without seasonality showed a better fit to the data compared to the model with seasonality; The [c]Durbin-Watson test does not directly provide a p-value but rather a test statistic (usually ranging from 0 to 4). Values close to 2 indicate no autocorrelation, while values near 0 suggest positive autocorrelation, and values near 4 suggest negative autocorrelation. The Durbin-Watson test suggests serial autocorrelation when the p-value is less than 0.05; [d]Autocorrelation terms defined by the autocorrelation function (ACF) and the partial autocorrelation function (PACF); e [e]DNA (Does not apply) means that both the Durbin-Watson test and the autocorrelation and partial autocorrelation functions did not indicate serial autocorrelation in the model residuals.
