# Supplementary material for: Effect of the COVID-19 Pandemic on Suicide Mortality in Brazil: An Interrupted Time Series Analysis
Source: Int J Environ Res Public Health. 2025 Jan 21;22(2):138. doi: 10.3390/ijerph22020138 (PMC11855535; doi:10.3390/ijerph22020138)
Supplement: Supplementary file 1 [file ijerph-22-00138-s001.zip › Table S2.pdf]

Table S2. Effect of the Covid-19 pandemic on suicide mortality rates per 100,000 women, estimated through interrupted time series analysis and by comparing the periods January 2017 to February 2020 with March 2020 to December 2023, Brazil, 2024

Variables	Behavior after the intervention	Initial exploratory model without serial autocorrelation terms			Final model with n serial autocorrelation terms		
		Coefficient	Standar Error	Durbin Watson test[c]	Coefficient	Standar Error	AR-terms[d]
Locality	Brazil[a]						
	Level change	-0.005	0.035	0.106	DNA[e]	DNA[e]	DNA[e]
	Trend change	0.004	0.001		DNA[e]	DNA[e]	DNA[e]
	North[b]						
	Level change	-0.071	0.097	0.8032	DNA[e]	DNA[e]	DNA[e]
	Trend change	0.006	0.002		DNA[e]	DNA[e]	DNA[e]
	Northeast[b]						
	Level change	0.036	0.066	0.386	DNA[e]	DNA[e]	DNA[e]
	Trend change	0.004	0.001		DNA[e]	DNA[e]	DNA[e]
	Southeast[a]						
	Level change	-0.071	0.062	0.455	DNA[e]	DNA[e]	DNA[e]
	Trend change	0.005	0.001		DNA[e]	DNA[e]	DNA[e]
	South[b]						
	Level change	0.049	0.044	0.403	DNA[e]	DNA[e]	DNA[e]
	Trend change	0.004	0.001		DNA[e]	DNA[e]	DNA[e]
	Midwest[b]						
	Level change	-0.083	0.091	0.238	DNA[e]	DNA[e]	DNA[e]
	Trend change	0.005	0.001		DNA[e]	DNA[e]	DNA[e]
Age groups (years)	10 to 14 years[b]						
	Level change	0.049	0.162	0.763	DNA[e]	DNA[e]	DNA[e]
	Trend change	0.003	0.003		DNA[e]	DNA[e]	DNA[e]
	15 to 19 years [b]						
	Level change	0.051	0.102	0.042	0.041	0.061	p=1
	Trend change	0.003	0.002		0.021	0.004	
	20 to 39 years[a]						
	Level change	-0.030	0.046	0.428	-0.033	0.005	p=3
	Trend change	0.007	0.001		0.007	0.000	
	40 to 59 years[b]						
	Level change	-0.031	0.055	0.171	DNA[e]	DNA[e]	DNA[e]
	Trend change	0.004	0.001		DNA[e]	DNA[e]	DNA[e]
	60 or more years[a]						
	Level change	0.044	0.078	0.1632	0.037	0.009	p=2
	Trend change	0.001	0.002		0.001	0.000	
Methods	Firearm[b]						
	Level change	0.088	0.125	0.301	DNA[e]	DNA[e]	DNA[e]
	Trend change	-0.003	0.003		DNA[e]	DNA[e]	DNA[e]
	Self-poisoning[b]						
	Level change	0.024	0.066	0.185	DNA[e]	DNA[e]	DNA[e]
	Trend change	0.004	0.000		DNA[e]	DNA[e]	DNA[e]
	Hanging, strangulation and suffocation[a]						
	Level change	-0.027	0.044	0.055	DNA[e]	DNA[e]	DNA[e]
	Trend change	0.005	0.001		DNA[e]	DNA[e]	DNA[e]

**Race/skin color**	White[a]						
	Level change	0.018	0.043	0.200	DNA[e]	DNA[e]	DNA[e]
	Trend change	0.003	0.001		DNA[e]	DNA[e]	DNA[e]
	Black[b]						
	Level change	-0.038	0.047	0.508	DNA[e]	DNA[e]	DNA[e]
	Trend change	0.007	0.001		DNA[e]	DNA[e]	DNA[e]

[a]Interrupted time series in which the quasi-Poisson model with seasonality showed a better fit to the data compared to the model without seasonality; [b]Interrupted time series in which the quasi-Poisson model without seasonality showed a better fit to the data compared to the model with seasonality; The [c]Durbin-Watson test does not directly provide a p-value but rather a test statistic (usually ranging from 0 to 4). Values close to 2 indicate no autocorrelation, while values near 0 suggest positive autocorrelation, and values near 4 suggest negative autocorrelation. The Durbin-Watson test suggests serial autocorrelation when the p-value is less than 0.05; [d]Autocorrelation terms defined by the autocorrelation function (ACF) and the partial autocorrelation function (PACF); e [e]DNA (Does not apply) means that both the Durbin-Watson test and the autocorrelation and partial autocorrelation functions did not indicate serial autocorrelation in the model residuals.
