# Supplementary figures and images for: Effect of the COVID-19 Pandemic on Suicide Mortality in Brazil: An Interrupted Time Series Analysis
Source: Int J Environ Res Public Health. 2025 Jan 21;22(2):138. doi: 10.3390/ijerph22020138 (PMC11855535; doi:10.3390/ijerph22020138)

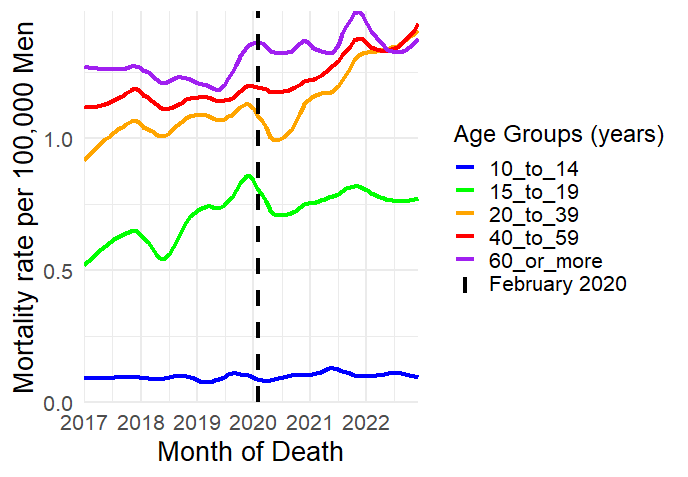

Supplement: Supplementary file 1 [file ijerph-22-00138-s001.zip › Figure S4. Smoothed monthly suicide rates per 100,000 men using LOESS by age group.tiff]

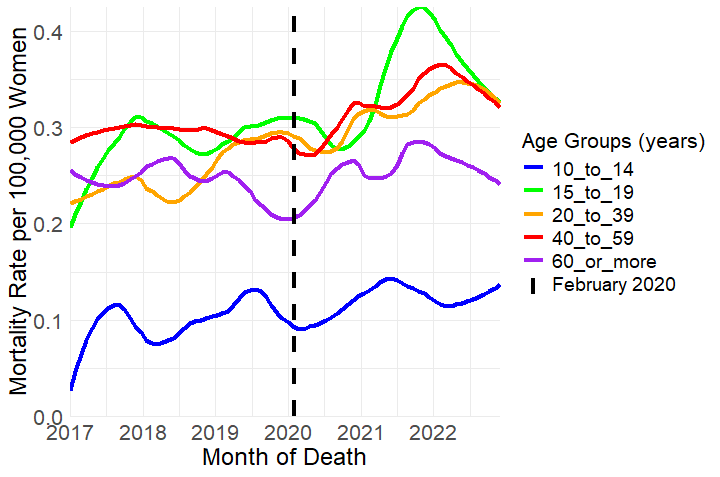

Supplement: Supplementary file 1 [file ijerph-22-00138-s001.zip › Figure S5. Smoothed monthly suicide rates per 100,000 women using LOESS by age group.png]

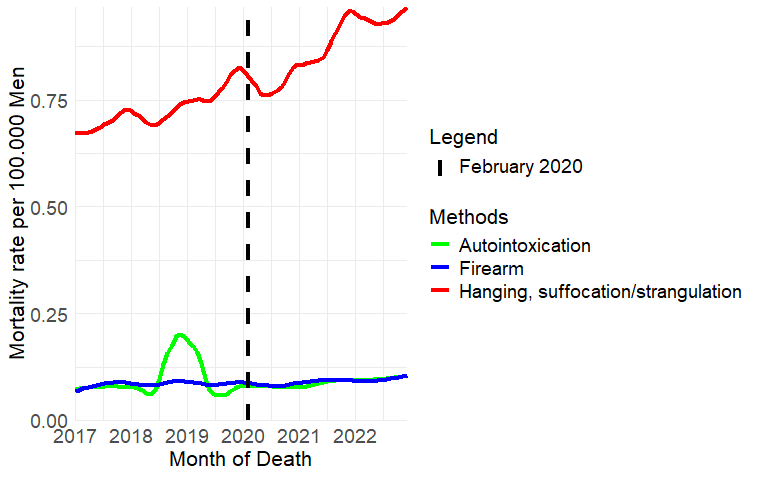

Supplement: Supplementary file 1 [file ijerph-22-00138-s001.zip › Figure S1. Smoothed monthly suicide rates per 100,000 men using LOESS by methods.tiff]

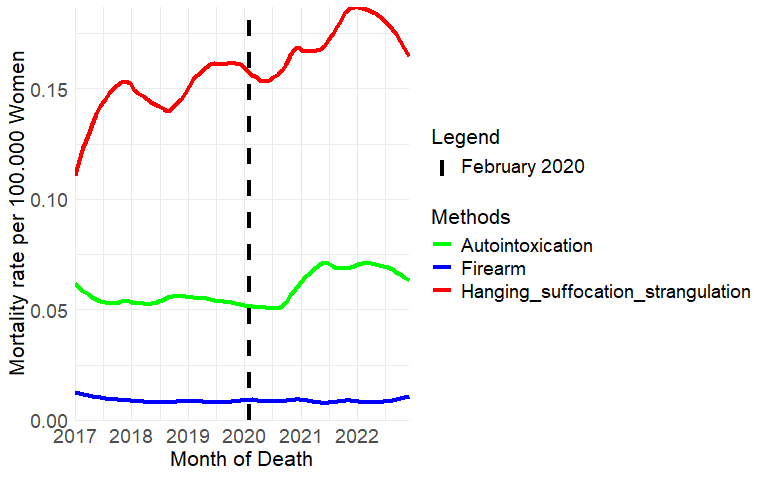

Supplement: Supplementary file 1 [file ijerph-22-00138-s001.zip › Figure S2. Smoothed monthly suicide rates per 100,000 women using LOESS by methods.png]

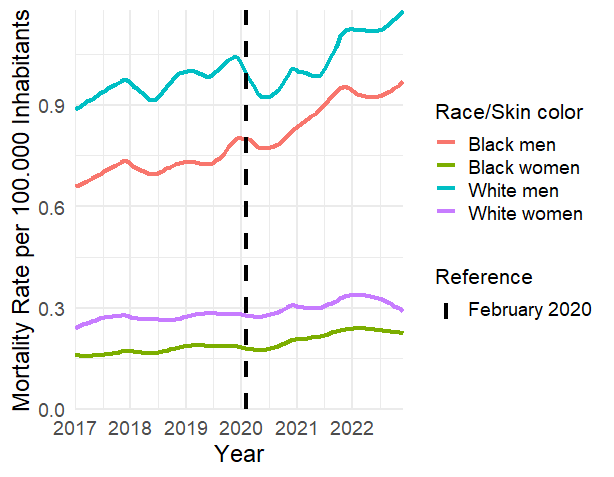

Supplement: Supplementary file 1 [file ijerph-22-00138-s001.zip › Figure S3. Smoothed monthly suicide rates per 100,000 inhabitants using LOESS by race and skin color.png]
